# The socio-professional impact of workaholism on engineers

**DOI:** 10.1192/j.eurpsy.2021.1506

**Published:** 2021-08-13

**Authors:** A. Hrairi, F. Dhouib, R. Masmoudi, N. Kotti, K. Jmal Hammami, M. Larbi Masmoudi, J. Masmoudi, M. Hajjeji

**Affiliations:** 1 The Department Of Occupational Medicine And Work-related Pathology, University hospital Hedi Chaker Sfax, SFAX, Tunisia; 2 Department Of Occupational Medicine, HEDI CHAKER hospital, SFAX, Tunisia; 3 Psychiatry A, hedi chaker hospital, Sfax, Tunisia; 4 Psychiatrie “a” Department, Hedi Chaker Hospital University -Sfax - Tunisia, sfax, Tunisia

## Abstract

**Introduction:**

Workaholism is an “irrational commitment to excessive work” as described by Cherrington. It’s considered as an emerging phenomenon that has been the topic of much debate. Indeed, over the last four decades, many contradictions have arisen among researchers investigating its negative consequences.

**Objectives:**

-Determine the prevalence of workaholism among a population of engineers. -Evaluate the socio-professional impact of workaholism on this population.

**Methods:**

This study is a descriptive-cross sectional analysis conducted on active engineers for one month. Data were collected through an online questionnaire, including socio-professional data and the WART (Work Addiction Risk Test) questionnaire.

**Results:**

Our population consisted of 75 engineers with an average age of 29± 4.6 years and sex-ratio of 1.2. Among this group, 26.7% of engineers were at risk of work addiction, while a certain addiction was noted among the third of the population. Workaholism was positively correlated with the lack of entertainment, especially sports activity (p= 0.012). Moreover, workaholic subjects were more likely to work more than 8 hours a day (p=0.004) and without a weekly break (p=0.043). Workaholism was not associated with the level of job satisfaction.

**Conclusions:**

Workaholism is an emerging phenomenon among engineers that can lead, in some cases, to depression and burnout. Therefore, the role of the occupational physician consists in the detection of early signs of workaholism and in raising awareness of this hidden problem.

**Conflict of interest:**

No significant relationships.

